# RT-PCR Misdiagnosis of Patient with Rare EGFR Mutation Lung Adenocarcinoma: Is NGS the Only Solution?

**DOI:** 10.3390/diagnostics15070842

**Published:** 2025-03-25

**Authors:** Piotr Piekarczyk, Urszula Lechowicz, Janusz Szopiński, Mateusz Polaczek, Katarzyna Błasińska, Katarzyna Modrzewska

**Affiliations:** 1 3rd Department of Lung Diseases and Oncology, National Tuberculosis and Lung Diseases Research Institute, 01-138 Warsaw, Poland; 2 Department of Genetics and Clinical Immunology, National Tuberculosis and Lung Diseases Research Institute, 01-138 Warsaw, Poland; 3 Department of Radiology, National Tuberculosis and Lung Diseases Research Institute, 01-138 Warsaw, Poland

**Keywords:** lung cancer, NGS, RT-PCR

## Abstract

**Background and Clinical Significance:** Molecular testing plays a crucial role in lung cancer diagnosis and management. While single-gene tests (SGTs) remain an important diagnostic tool, developments in novel methods such as next generation sequencing (NGS) provide a more precise mutational profile and enable the targeted treatment of a larger scope of mutation-driven cancers. **Case presentation:** We present a case of a patient with a rare *EGFR* variant lung adenocarcinoma, who was misdiagnosed using a SGT. The initial treatment with immunotherapy was unsuccessful. **Conclusions:** The patient could have benefited if NGS had been performed instead of traditional real-time polymerase chain reaction (RT-PCR) and if adequate tyrosine kinase inhibitor treatment was initiated at the time of diagnosis.

## 1. Introduction

Lung cancer remains a leading cause of cancer-related mortality worldwide, with non-small-cell lung cancer (NSCLC) representing the most common histological subtype. The advent of targeted therapies has transformed treatment paradigms for patients with NSCLC harboring specific driver variants. The development and widespread use of novel drugs targeting specific driver variants in lung cancer, such as the epidermal growth factor receptor (*EGFR*), anaplastic lymphoma kinase (*ALK*), and ROS proto-oncogene 1, receptor tyrosine kinase (*ROS1*), have revolutionized treatment approaches and have become a critical step in the management of NSCLC. According to the National Comprehensive Cancer Network Guidelines Version 1.2025 for NSCLC, there are more than eleven genes for which there are targeted therapies, and accurate and timely molecular profiling allows for the selection of patients who are most likely to benefit from targeted therapies, while sparing those without these variants from unnecessary treatments and potential side effects. Thus, identifying actionable variants in lung cancer diagnostic workup has become a crucial step in accurate cancer diagnosis, guiding physicians towards personalized treatment plans [1,2,3].

While real-time polymerase chain reaction (RT-PCR) remains a valuable tool, next generation sequencing (NGS) technology’s superior capabilities position it as a powerful and evolving force in lung cancer diagnosis, paving the way for more precise diagnoses and individualized treatment approaches. Traditionally, RT-PCR has been the mainstay for detecting *EGFR* variants in NSCLC. However, this technique has inherent limitations, including limited variant detection (potentially missing relevant alterations) due to focusing on specific and/or restricted number of variants, thus overlooking rare or novel occurrences, and limited sensitivity for detecting low-frequency variants or those present in small tumor populations.

These limitations highlight the need for more comprehensive and sensitive diagnostic approaches. NGS has emerged as a powerful tool for overcoming these challenges. NGS technology offers several advantages over conventional methods, including: (i) high sensitivity and specificity (NGS platforms can detect low-frequency variants and identify a broader spectrum of genetic alterations), (ii) simultaneous detection of multiple genes (NGS enables the simultaneous analysis of multiple genes, including both known driver variants and emerging biomarkers), (iii) potential for novel discovery (NGS can uncover novel variants and biomarkers, paving the way for future therapeutic advancements). While NGS offers significant advantages, it is important to consider the ethical implications of using this technology, such as the potential for incidental findings and the need for appropriate genetic counseling.

We will discuss the patient’s clinical presentation, diagnostic journey, and how the patient could have benefited from NGS as a first-line test (if it were available at the time of first molecular testing) if it had been performed instead of RT-PCR. This case highlights the importance of NGS as a first-line diagnostic approach in the era of precision oncology.

## 2. Case Report

A 62-year patient, diagnosed with lung adenocarcinoma after supraclavicular lymph node biopsy, was admitted to hospital. He had a history of deep vein thrombosis, hypertension, benign prostatic hyperplasia and smoking (20 packyears; he quit 20 years ago). Thoracic computed tomography (CT) performed prior to hospital admission revealed a 46 × 47 mm mass in the second and sixth segments of the left lung, with multiple metastases in both lungs, with bilateral supraclavicular, subcarinal and hilar lymphadenopathy.

On admission, the patient was in performance status 1 of the Eastern Cooperative Oncology Group (ECOG) scale. He complained of dyspnea on exertion, and dry cough for two months. He lost 4 kg of weight in six months. On auscultation, he had basilar crackles on the left side. A diagnostic workup of predictive biomarkers of patient’s histopathological specimens was ordered.

**Figure 1:**
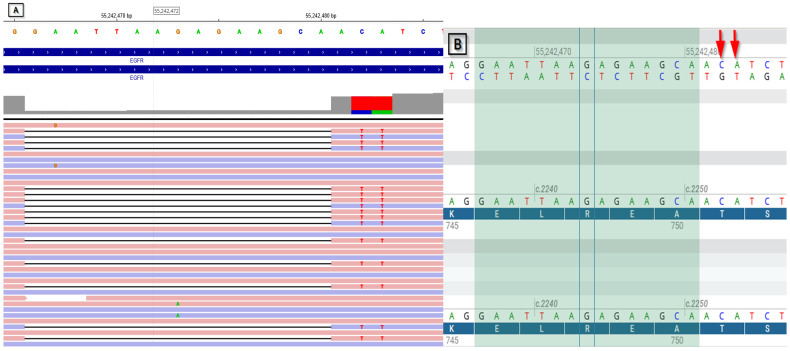
Picture presenting the EGFR NM_005228.5:c.2236_2253delinsATT variant. Specifically, (**A**) shows the variant as displayed in Archer Analysis 7.2.1, and (**B**) visualizes the deletion region in the wild-type sequence and the substitutions using Alamut Visual Plus v. 1.12.; pictures explanation: (**A**,**B**) A- adenine, T- thymine, G—guanine, C—cytosine; (**A**) purple lines—read strand (−), pink lines- read strand (+), black lines—indicate the deletion; (**B**) highlighted region- region of the deletion; red arrows—indicate the place of substitution of C and A with T.

A diagnosis of adenocarcinoma was confirmed (CKAE/AE3+, CK7+, TTF-1+). Formalin-fixed, paraffin-embedded (FFPE) tissue section was used to perform DNA extraction with the spin column-based cobas DNA sample preparation kit (Roche Molecular Systems, Inc., South Branchburg, NJ, USA). The sample was tested for *EGFR* variants using the cobas *EGFR* mutation test v2 assay (Roche Molecular Systems). Immunohistochemistry for *ALK* protein expression was performed using VENTANA ALK (CloneD5F3). Fluorescence in situ hybridization (FISH) was carried out on FFPE sections using the ZytoLight FISH-Tissue Implementation Kit (ZytoVision GmbH, Bremerhaven, DE-HB, Germany) and Spec *ROS1* Dual Color Break Apart Probe (ZytoVision GmbH). No clinically relevant variants were found in any of these tests. PD-L1 was assessed using Ventana antibody, revealing expression on 20% of tumor cells (TPS). Thoracic, abdominal, and pelvic CT with contrast was performed according to RECIST 1.1 criteria. The target lesions were as follows: tumor in left hilum 46 mm, right hilar lymph nodes 47 mm and celiac lymph node 17 mm, and sum of length diameter (SLD) 110 mm. The nontarget lesions were as follows: enlarged subcarinal lymph nodes; upper and lower paratracheal lymph nodes; multiple nodules in both lungs; and radiological features of lymph vessels invasion, namely ground glass opacities, septal thickening, and small nodules with perilymphatic distribution. Moreover, a subsegmental pulmonary embolism was revealed. The patient was started on an anticoagulant. Cranial CT was also performed, revealing no abnormalities. The disease clinical stage was initially classified as IVA (cT2bN3M1a) by 8th Edition of TNM UICC. He commenced immunochemotherapy with a regimen of 4 induction courses of cisplatin, pemetrexed, pembrolizumab, and a continuation with pembrolizumab and subsequently pemetrexed. A thoracic CT scan was performed after 3 months of treatment, revealing a partial response (SLD 63 mm). The treatment was continued, with a total of 11 courses. Subsequent CT scans, which were carried out every 3 months, showed a partial response (SLD 63 mm) and progressive disease (SLD 76 mm), accordingly. The treatment was discontinued. It was decided that further diagnostic steps must be taken to reassess the patient. Ultrasound-guided supraclavicular biopsy was performed and newly implemented RNA–based targeted NGS analysis with FusionPlex^®^ Lung v2 (Integrated DNA Technologies (IDT), Inc., Coralville, IA, USA) was conducted. Libraries were paired-end-sequenced in 2 × 150 cycles on an Illumina MiSeq instrument (Illumina, Inc., San Diego, CA, USA) using the high-output MiSeq Reagent Kit v3 (Illumina). Data were analyzed using the Archer Analysis v.7.2.1 software (Archer/IDT) for the presence of gene fusion, using GRCh37 as the reference genome. An *EGFR* exon 19 variant was detected (Figure 1). This variant involves an in-frame deletion of amino acids in positions 746–751 and the insertion of isoleucine (NM_005228.5:c.2236_2253delinsATT p.Glu746_Thr751delinsIle), and is presented in Figure 1. No other clinically relevant variants, such as *ALK*; *NTRK1*; *NTRK2*; *NTRK3*; *RET*; *ROS1* fusions; *MET* exon 14 skipping; *BRAF*, *ERBB2*, *KRAS* single-nucleotide polymorphisms; or insertions/deletions were observed.

The patient started a treatment of 150 mg of erlotinib daily.

**Figure 2:**
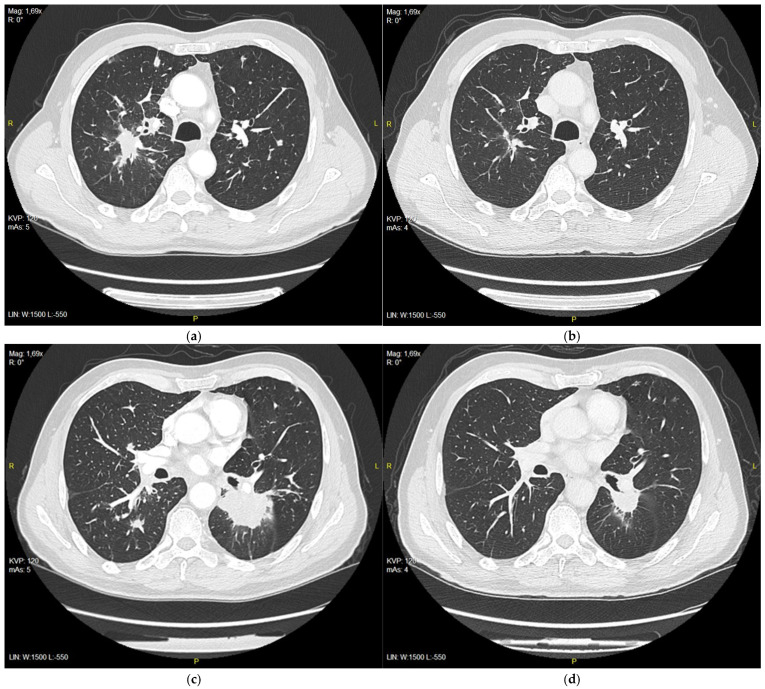
(**a**) Right upper lobe nodule at the initiation of the treatment with erlotinib, 28 mm in diameter; (**b**) right upper lobe nodule after 3 months of treatment, 11 m in diameter; (**c**) tumor in 6th segment of left lung at the initiation of the treatment with erlotinib, 51 mm in diameter; (**d**) tumor after 3 months of treatment, 25 mm in diameter.

After 3 months of treatment, CT evaluation was performed by RECIST 1.1 and a partial response was observed, with a reduction in target lesions from 111 mm to 59 mm (Figure 2a–d).

**Figure 3:**
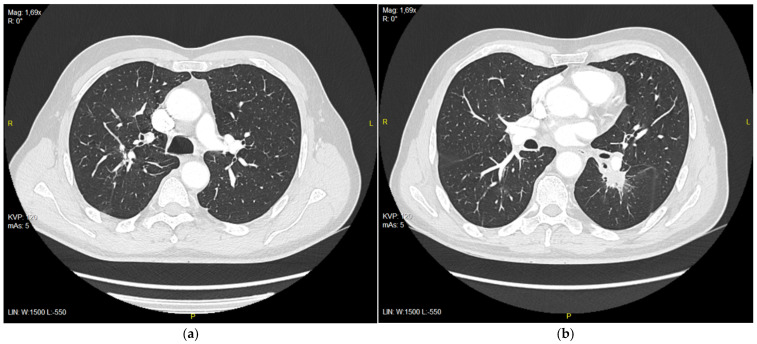
(**a**) Right upper lobe nodule after 9 months of treatment with erlotinib, 9 mm in diameter; (**b**) tumor in 6th segment of left lung after 9 months of treatment with erlotinib, 21 mm in diameter.

The patient continued on the treatment with good clinical effects. A partial response was observed on CT studies performed after 3, 6, and 9 months (Figure 3 and Table 1).

## 3. Discussion

The early and accurate diagnosis of lung cancer is crucial for optimal treatment outcomes and improved patient survival. NGS can facilitate rapid and comprehensive molecular profiling, enabling clinicians to make timely and informed treatment decisions. NGS enables personalized medical treatment by identifying targetable variants and selecting the most appropriate treatment options for each individual patient, and can be used to refine risk stratification and to monitor treatment response. This approach led to improved treatment outcomes and reduced side effects.

Our case highlights the importance of comprehensive molecular testing among patients with lung cancer. Real-time PCR, while commonly used, is limited to detecting the specific variants included in the kit design and may have troubles with detecting certain variants, especially uncommon or complex ones [4,5,6]. Conversely, NGS analyzes a broader range of genes, enabling the identification of less common and more complex variants, potentially leading to improved therapeutic decision-making. By providing a more comprehensive understanding of the patient’s tumor genetics, NGS can help clinicians select the most appropriate treatment options and optimize patient care. Our case exemplifies this point. Exon 19 in-frame deletions constitute two-thirds of 85–95% of druggable EGFR variants, and should be detected by all diagnostic platforms [7]. The cobas EGFR Mutation Test v2 detects 2236_2253del18 deletion; however, the specific variant observed in our patient, characterized by an in-frame deletion accompanied by an isoleucine insertion (c.2236_2253delinsATT), likely hindered detection by this PCR-based kit. Although this variant of EGFR is rare, it has been previously described in a case report with response to TKI treatment (with gefitinib) [8]. Our patient also benefited from treatment with erlotinib, clearly indicating a link between the abovementioned variant and the sensitivity of patients’ lung adenocarcinoma in this kind of therapy.

Initially, our patient received a standard treatment on the basis of his molecular profile (after the exclusion of *EGFR, ALK, ROS1* mutations). While NGS provides an in-depth analysis of those biomarkers, ESMO guidelines point to several methods for ruling out the presence of druggable molecular targets: real-time PCR or NGS for *EGFR* variants and IHC, FISH, or RNA-based NGS for *ALK* variants. Neither pembrolizumab clinical trials nor the summary of European Medicine Agency’s products characteristics of pembrolizumab recommend any particular tests that must be used. As it occurred in the case of our patient, rare variants can be overlooked by using a basic molecular test workup that does not include NGS. This can lead to worse clinical outcomes as immunotherapy is not generally considered a recommended treatment option in the presence of druggable variants in *ALK* and *EGFR* genes, and its use even in the third or fourth lines remains controversial. The only recommended regimen according to ESMO guidelines is atezolizumab–bevacizumab–paclitaxel–carboplatin, which may be considered after EGFR TKI failure.

NGS is the preferred method for molecular diagnostics of several genomic alterations according to the latest ESMO guidelines. It surpasses RT-PCR in several key areas as a diagnostic tool, like facilitating the identification of novel variants and multiplexing (NGS can simultaneously analyze hundreds of samples and a vast array of genes, while real-time PCR typically focuses on a limited number of known variants) [9]. Moreover, NGS gives the possibility of comprehensive pathogenic variants analysis, and beyond identifying specific variants, NGS delves deeper by providing information on the type and location of the mutation, including the allele frequency (percentage of cells harboring the mutation) [10].

Clinically, this means that, firstly, it requires less tissue compared to SGT when analyzing more than four biomarkers (almost always a case in lung cancer). Secondly, it provides a more precise analysis of targeted genes. Studies have shown that NGS can detect approximately 50% more insertions and duplications in exon 20 of the *EGFR* gene compared to PCR. Additionally, NGS facilitates the analysis of gene fusions (*ALK*, *ROS1*, *NTRK1/2/3*, *RET*) alongside point variants, which hold significant implications for targeted therapy selection with tyrosine kinase inhibitors (TKIs) [11]. Other clinically relevant variants are mainly located in *KRAS*, *BRAF*, *HER2*, *PIK3CA* and *MET* genes [12]. Finally, NGS can be cost-effective compared to sequential SGT. Research suggests that upfront NGS analysis in all lung cancer patients could streamline the diagnostic process, optimize targeted therapy allocation, and potentially reduce the number of undiagnosed and ineffectively treated patients [11].

While the survival benefit of using NGS compared to SGT as a diagnostic tool to implement molecular treatment in oncogene-addicted NSCLC in real-world settings remains under investigation, several studies have explored their effectiveness. Kang et al. analyzed data from 8566 patients with advanced lung adenocarcinoma and concluded that upfront NGS testing did not demonstrably improve survival outcomes of oncologic treatment compared to SGT for ALK and EGFR testing [13]. While NGS was associated with improved survival in unadjusted analyses, this association disappeared after adjusting for confounding variables or employing matched cohort designs. The authors hypothesized that this might be attributed to favorable socioeconomic factors in the NGS group, such as younger age, higher income, and easier access to healthcare and immunotherapy. In their analysis of 5688 patients, similar findings were reported by Presley et al., although the author pointed out financial barriers that may have hindered the availability of targeted treatment among the patients, thus not impacting their survival [14].

Currently NGS is primarily used to administer guided treatment in variant driven NSCLC but its potential use extends to the assessment of prognosis and responsiveness to targeted therapies. It is unclear why certain patients do not benefit from treatment with TKIs and it may be connected to concomitant gene variants. Although the knowledge is limited at this time, there are a number of studies that assessed responsiveness to EGFR, ALK, and ROS1 TKIs in the presence of *TP53* variants that showed negative effects on treatment response. Moreover, there was a difference in PFS on the basis of the type of variants. This could also guide further therapeutic decisions, as some studies showed benefits of using anti-angiogenic drug or chemotherapy with EGFR-TKI vs. EGFR-TKI plus placebo in *EGFR*- and *TP53*-positive NSCLC [15,16]. A study by Zhao et al. showed longer PFS in the group receiving gefitinib plus apatinib vs. in the gefitinib plus placebo group (12.5 vs. 1.1 months, respectively; HR 0.56) in a cohort of patients with exon 8 *TP53* variant [17]. Similar results were demonstrated by the RELAY trial where the group treated with ramucirumab plus erlotinib vs. erlotinib alone had a PFS o 15.2 months compared to 10.6 months (HR 0.54). For a combination of EGFR-TKI plus chemotherapy or an anti-angiogenic drug vs. EGFR-TKI alone in a group of patients with *TP53* exon 4/7 mutation, Sun et al. presented that the median PFS was significantly longer in the combined therapy group (18 vs. 7 months) [18]. Additionally, NGS can facilitate the enrollment of patients into targeted clinical trials based on their specific genetic profiles. This can accelerate the development of new therapies and improve access to innovative treatments for patients with lung cancer.

NGS upfront testing is still not a standard in many laboratories [19]. When comparing different methods for the molecular diagnosis of NSCLC, the main factor that may impede broader NGS adoption is its cost. However, in their study, Seminati et al. performed an economic analysis employing a full-cost approach, encompassing direct and indirect costs, overheads, VAT (value-added tax) in Italy and estimated that a comprehensive cost for each sample amounts to EUR 1048.32 and is estimated at 1% of all the expenses connected to diagnostic and therapeutic interventions [20]. Inadequate diagnosis, as in the case of our patients, can increase the cost by administering inadequate treatment that may lead to a greater economic burden on the healthcare system than upfront NGS testing.

Some authors propose upfront SGT testing followed by NGS. Mary K Nesline et al. considered the impact of such strategy on outcomes of subsequent comprehensive genomic profiling (CGP) in NSCLC [21]. In their work, they contacted community-based oncologists who ordered SGT to assess predictive biomarkers and offered them CGP (DNA and RNA sequencing). Only 3% of orders for SGTs included tests for all recommended genes. A total of 561 patients were identified, among which 135 had an initial negative combination of SGTs and subsequently had CGP performed. In 46% of cases, patients had a positive result for one of the recommended biomarkers. Moreover, this group experienced more GCP cancelations due to an insufficient amount of tissue for analysis (17% vs. 7%), turnaround time > 14 days (62% vs. 29%) and DNA sequencing failures (13% vs. 8%) than the group with initial CGP testing.

The benefits of using NGS are very clear in the context of palliative treatment in metastatic lung cancer, as in the case of our patient. However, contemporary treatment of locally advanced lung cancer requires a different approach. In the era of perioperative treatment with immunochemotherapy, a quick turnaround time for a few genetic tests is sometimes more important than the precise analysis of all possible clinically relevant variants. As mentioned above, NGS turnaround time is better than the sequential analysis of many genes. However, the administration of immunotherapy only requires the exclusion of EGFR and ALK variants (and also ROS1 in some countries). In those cases, SGTs are better as first-line tests as they allow for very rapid detection of the most common pathogenic variants in these genes [4]. In earlier stages, time is of the essence, as every delay of appropriate treatment can lead to the progression of the disease, loss of resectability, and thus worse outcomes.

Despite the ongoing exploration of survival benefits, compelling arguments support the adoption of NGS as the first-line diagnostic tool for lung cancer patients. While NGS may not yet be the standard of care for all lung cancer patients, its increasing accessibility and growing body of evidence support its potential to improve diagnostic accuracy and treatment outcomes. Its comprehensive nature and potential to identify a broader spectrum of mutations hold promise for optimizing therapeutic strategies and improving patient outcomes [22,23,24]. In this case, our patient could have been given a different treatment from the beginning, which would have potentially improved his prognosis.

## 4. Conclusions

NGS is a precise method of molecular testing which provides more insight into a patient’s mutational profile compared to RT-PCR.

## Figures and Tables

**Table 1:** Table presenting CT results evaluated according to RECIST 1.1 criteria at the start of the treatment and in 3-month intervals.

		Initial CT	CT After 3 Months	CT After 6 Months	CT After 9 Months
Target lesions	Perihilar tumor in 6th segment of the left lung	51 mm	25 mm	28 mm	21 mm
	Tumor in the right upper lobe on the border of 1st and 2nd segments	28 mm	11 mm	14 mm	9 mm
	Right hilar lymph nodes	18 mm	14 mm	14 mm	11 mm
	Celiac trunk lymph node	14 mm	9 mm	7 mm	7 mm
	Sum of target lesions	111	59	63	48
Non-target lesions	Subcarinal lymph nodes	Lymphadenopathy	Normal	Normal	Normal
	Irregular patchy nodular metastatic infiltrates in both lungs	Present	Present; reduced in number and size	Present	Present; further reduction
	Interlobular septal thickening—dissemination via lymphatic vessels	Present	Present; minimal	Present; minimal	Present; minimal

## Data Availability

The original contributions presented in this study are included in the article. Further inquiries can be directed to the corresponding author.

## References

[1] Ramalingam S.S., Vansteenkiste J., Planchard D., Cho B.C., Gray J.E., Ohe Y., Zhou C., Reungwetwattana T., Cheng Y., Chewaskulyong B. (2020). Overall Survival with Osimertinib in Untreated EGFR-Mutated Advanced NSCLC. N. Engl. J. Med..

[2] Peters S., Camidge D.R., Shaw A.T., Gadgeel S., Ahn J.S., Kim D.W., Ou S.H.I., Pérol M., Dziadziuszko R., Rosell R. (2017). Alectinib versus Crizotinib in Untreated ALK-Positive Non-Small-Cell Lung Cancer. N. Engl. J. Med..

[3] Shaw A.T., Riely G.J., Bang Y.J., Kim D.W., Camidge D.R., Solomon B.J., Varella-Garcia M., Iafrate A., Shapiro G., Usari T. (2019). Crizotinib in ROS1-rearranged advanced non-small-cell lung cancer (NSCLC): Updated results, including overall survival, from PROFILE 1001. Ann. Oncol..

[4] Colling R., Bancroft H., Langman G., Soilleux E. (2019). Fully automated real-time PCR for EGFR testing in non-small cell lung carcinoma. Virchows Arch..

[5] Torres S., González Á., Cunquero Tomas A.J., Calabuig Fariñas S., Ferrero M., Mirda D., Sirera R., Jantus-Lewintre E., Camps C. (2020). A profile on cobas® EGFR Mutation Test v2 as companion diagnostic for first-line treatment of patients with non-small cell lung cancer. Expert. Rev. Mol. Diagn..

[6] Batra U., Nathany S., Sharma M., Jain P., Mehta A. (2022). Next generation sequencing for detection of EGFR alterations in NSCLC: Is more better?. J. Clin. Pathol..

[7] Imyanitov E.N., Iyevleva A.G., Levchenko E.V. (2021). Molecular testing and targeted therapy for non-small cell lung cancer: Current status and perspectives. Crit. Rev. Oncol. Hematol..

[8] Smits A.J.J., Roepman P., Claessens N.J.M., Schramel F.M., Kummer A.J. (2016). Lung adenocarcinomas: A novel KRAS/EGFR exon 21 double mutation with limited response to TKI treatment and two rare EGFR exon 19 deletions with variable response. Int. Clin. Pathol. J..

[9] Gao J., Wu H., Shi X., Huo Z., Zhang J., Liang Z. (2016). Comparison of Next-Generation Sequencing, Quantitative PCR, and Sanger Sequencing for Mutation Profiling of EGFR, KRAS, PIK3CA and BRAF in Clinical Lung Tumors. Clin. Lab..

[10] Fernandes M.G.O., Jacob M., Martins N., Moura C.S., Guimarães S., Reis J.P., Justino A., Pina M.J., Cirnes L., Sousa C. (2019). Targeted Gene Next-Generation Sequencing Panel in Patients with Advanced Lung Adenocarcinoma: Paving the Way for Clinical Implementation. Cancers.

[11] Stencel K., Wasąg B., Pruszko C., Dąbrowska K., Książek P., Dziadek K., Bryl M., Krzakowski M. (2023). Clinical and economic benefits of using next-generation sequencing (NGS) in the diagnostics of patients with non-small cell lung cancer with rare mutations. Oncol. Clin. Pract..

[12] Cainap C., Balacescu O., Cainap S.S., Pop L.A. (2021). Next Generation Sequencing Technology in Lung Cancer Diagnosis. Biology.

[13] Kang D.W., Park S.K., Yu Y.L., Lee Y., Lee D.H., Kang S. (2024). Effectiveness of next-generation sequencing for patients with advanced non-small-cell lung cancer: A population-based registry study. ESMO Open..

[14] Presley C.J., Tang D., Soulos P.R., Chiang A.C., Longtine J.A., Adelson K.B., Herbst R.S., Zhu W., Nussbaum N.C., Sorg R.A. (2018). Association of Broad-Based Genomic Sequencing with Survival Among Patients With Advanced Non-Small Cell Lung Cancer in the Community Oncology Setting. JAMA.

[15] Moes-Sosnowska J., Szpechcinski A., Chorostowska-Wynimko J. (2024). Clinical significance of TP53 alterations in advanced NSCLC patients treated with EGFR, ALK and ROS1 tyrosine kinase inhibitors: An update. Tumour Biol..

[16] Mehta A., Ghosh Mitra A., Mani S., Dewan H., Mattoo S., Batra U. (2025). Prognosis of TP53 and Its Concomitant EGFR Mutation in Lung Cancer Especially Non-Small Cell Lung Cancer. Asian Pac. J. Cancer Prev..

[17] Zhao H., Yao W., Min X., Gu K., Yu G., Zhang Z., Cui J., Miao L., Zhang L., Yuan X. (2021). Apatinib Plus Gefitinib as First-Line Treatment in Advanced EGFR-Mutant NSCLC: The Phase III ACTIVE Study (CTONG1706). J. Thorac. Oncol..

[18] Sun H., Ren P., Chen Y., Lan L., Yan Z., Yang Y., Wang B., Wang C., Li Y., Li L. (2023). Optimal therapy for concomitant EGFR and TP53 mutated non-small cell lung cancer: A real-world study. BMC Cancer.

[19] Hofman P., Calabrese F., Kern I., Adam J., Alarcão A., Alborelli I., Anton N., Arndt A., Avdalyan A., Barberis M. (2023). Real-world EGFR testing practices for non-small-cell lung cancer by thoracic pathology laboratories across Europe. ESMO Open..

[20] Seminati D., L’Imperio V., Casati G., Ceku J., Pilla D., Scalia C.R., Gragnano G., Pepe F., Pisapia P., Sala L. (2024). Economic assessment of NGS testing workflow for NSCLC in a healthcare setting. Heliyon.

[21] Nesline M.K., Subbiah V., Previs R.A., Strickland K.C., Ko H., DePietro P., Biorn M.D., Cooper M., Wu N., Conroy J. (2024). The Impact of Prior Single-Gene Testing on Comprehensive Genomic Profiling Results for Patients with Non-Small Cell Lung Cancer. Oncol. Ther..

[22] de Jager V.D., Timens W., Bayle A., Botling J., Brcic L., Büttner R., Fernandes M.G.O., Havel L., Hochmair M., Hofman P. (2024). Future perspective for the application of predictive biomarker testing in advanced stage non-small cell lung cancer. Lancet Reg. Health Eur..

[23] Kerr K.M., Bibeau F., Thunnissen E., Botling J., Ryška A., Wolf J., Öhrling K., Burdon P., Malapelle U., Büttner R. (2021). The evolving landscape of biomarker testing for non-small cell lung cancer in Europe. Lung Cancer.

[24] Batra U., Nathany S. (2025). Biomarker testing in lung cancer: From bench to bedside. Oncol. Rev..

